# Traditional Maasai Dietary Practices and Their Inapplicability to Modern Carnivore Diets: A Narrative Review

**DOI:** 10.7759/cureus.78448

**Published:** 2025-02-03

**Authors:** David M Goldman, Thomas J Waterfall, Matthew Nagra

**Affiliations:** 1 Research and Development, Metabite, New York, USA; 2 Public Health, University of Helsinki, Helsinki, FIN; 3 Nutrition, Game Changers Institute, Laguna Niguel, USA; 4 Family Practice, University of British Columbia, Vancouver, CAN

**Keywords:** carnivore diet, diet and cardiovascular health, dietary generalizability, maasai diet, meat-based diet, pastoralist nutrition

## Abstract

The traditional dietary practices of the Maasai people frequently are cited to support meat-based diets in industrialized populations, owing to the historically low prevalence of cardiovascular disease among this nomadic pastoralist group. However, such comparisons typically neglect the multifaceted interplay of genetic, environmental, and lifestyle factors that underpin Maasai health outcomes. This narrative review critically examines the socio-ecological context of the Maasai, highlighting their unique genetic adaptations for cholesterol metabolism, high physical activity levels, intermittent fasting, calorie restriction, and high-altitude living. It also addresses the confounding effects of infectious diseases and a reduced life expectancy, which shape their cardiovascular risk profile. Significant differences in the dietary composition and context exist between the traditional Maasai diet and modern meat-based dietary patterns, rendering generalizations problematic. This review emphasizes the importance of population-specific factors and underscores the limitations of extrapolating health benefits attributed to the traditional Maasai diet to other populations who do not share these factors.

## Introduction and background

Dietary patterns play a crucial role in human health, and interest in low-carbohydrate, carnivorous diets for metabolic regulation, weight management, and chronic disease prevention has grown in recent years. Proponents frequently cite the traditional dietary practices of the Maasai people as evidence of the purported health benefits of meat-based diets [1-7]. The Maasai are semi-nomadic pastoralists primarily inhabiting southern Kenya and northern Tanzania, whose livelihoods center on herding cattle, sheep, and goats [8]. Unlike hunter-gatherer societies that rely on wild resources, the Maasai practice seasonal migration to manage grazing lands and water resources, establishing temporary settlements during the wet season and more permanent dwellings in the dry season [9]. Hypertension and hyperlipidemia are currently prevalent among the Maasai [10-12], and deficiencies in vitamins A, C, D, and B12 as well as folate and iron are common [13-15]. However, the historically low incidence of cardiovascular diseases (CVD) among the Maasai is often presented as justification for adopting similar dietary patterns in regions facing high rates of diet and lifestyle-related chronic diseases. Such interpretations may oversimplify and decontextualize the health outcomes of the Maasai by overlooking critical factors that collectively influence their health and complicate direct comparisons with other populations facing different circumstances.

The cardiovascular health of the Maasai reflects a complex interplay of dietary practices, genetics, and environmental influences. Although their traditional diet is high in saturated fat and cholesterol, the Maasai have genetic adaptations that enable efficient cholesterol metabolism and regulation, protecting them from hypercholesterolemia and related outcomes [16]. Furthermore, calorie restriction, intermittent fasting, copious physical activity, prevalent infections, and a high-altitude environment further influence CVD risk factors [17-21]. However, these factors do not shield the Maasai from significant health challenges, such as high rates of anemia and premature mortality [22-24].

This narrative review aims to critically evaluate the health implications of the Maasai diet and lifestyle by exploring the broader biological and environmental determinants. Their traditional, rather than current dietary patterns and lifestyle factors will be discussed because government-supported ranching programs implemented in the 1960s and 1970s resulted in a transition from the traditional diet, consisting largely of blood, milk, and meat to greater consumption of processed foods including refined grains and sugar [25]. This transition resulted in a 14-19% increase in calories, increased smoking and alcohol use, and reduced physical activity levels, which contributed to a 2 kg/m^2^ increase in body mass index (BMI) and increased prevalence of glucose intolerance or diabetes [25]. An examination of the current Maasai diet, lifestyle, and health outcomes would therefore be less relevant and unable to address the protective associations presented in more historical literature. By contextualizing the Maasai within their previous, unique socio-ecological framework, this review investigates the appropriateness of citing their traditional dietary practices in support of modern-day meat-based diets in industrialized countries. This will contribute to a nuanced understanding of the complex interplay between diet, lifestyle, and health.

The literature review was confined to studies examining the traditional Maasai diet, physical activity levels, environmental influences, and genetic factors, and their associations with cardiovascular health outcomes. Peer-reviewed research published in English from January 1, 1930 to January 1, 2024 was identified using PubMed and Google Scholar. Search terms included the following: Maasai diet, pastoralist nutrition, cholesterol metabolism, saturated fat intake, CVD, LDL, HDL, triglycerides, blood pressure, body weight, BMI, physical activity, calorie intake, intermittent fasting, genetic adaptations, infectious disease, and high-altitude living. When relevant, additional references cited within the identified studies were also reviewed. Priority was given to historical data that preceded major shifts in diet and lifestyle factors that began prior to 1980.

## Review

Traditional dietary intake

The traditional Maasai diet has been characterized by its reliance on animal products and minimal inclusion of plant-based foods. Biss et al. (1971) reported that milk was the primary dietary staple, with men drinking 3-5 liters per day. When milk supplies dwindled for 4-5 months per year during dry seasons, the Maasai supplemented their diet with fresh blood mixed into milk. They also consumed large quantities of roasted or boiled meat from cattle, sheep, or goats during these times, with meals estimated to include 1.8-2.3 kg of meat in a single sitting and 2-5 kg of meat on days when cattle are brought to market as well as celebration days [24,26,27]. Nestel (1989) reported that meat was less frequently consumed by women and children, typically 2-5 times per month, depending on the season and livestock availability. Non-pregnant and lactating women obtained 52% of their daily energy intake from dairy, and maize became an alternative staple when milk was scarce. However, fruits, vegetables, and pulses were rarely eaten [28]. This is largely due to beliefs that land is exclusively meant for grazing, which limits crop production, and that vegetables are livestock feed rather than human consumption [29].

Nutrient intake among the Maasai indicates that their traditional diet is high in fat and protein and low in carbohydrates. Early reports indicated that adult Maasai men and women consumed between 2,500 and 3,000 kcal/day, with 33-47% of energy derived from fat, 27-40% from protein, and 13-40% from carbohydrates [30]. Subsequent investigations indicated higher fat intakes (66-67% of total calories) and mean cholesterol intakes of 500-2,000 mg/day [26,28]. The mean fatty acid compositions in the diets of adults have been reported to be approximately 68%, 28%, and 4% saturated, monounsaturated, and polyunsaturated, respectively [31]. The traditional Maasai diet has also been reported to be devoid of added salt, meeting requirements through the consumption of animal foods [24]. Micronutrient analyses indicated that iron and vitamin C intakes were low, reflected by anemia prevalence of 39% of children under five years of age and 22% in adult women [22]. These findings depict the unique nutritional profile of the Maasai, which reflects their cultural practices while highlighting important gaps in micronutrient intake.

Cardiovascular risk and life expectancy

The Maasai present a complex cardiovascular health profile influenced by unique biological and lifestyle factors. Mann et al. (1964) conducted a cross‐sectional study involving 400 Maasai adolescent boys and men, aged 14 to >55 years, who were recruited from several communities in the region. The study used standardized clinical protocols: participants were examined after a resting period, and blood pressure was measured using a mercury sphygmomanometer with duplicate readings taken to ensure accuracy. Hypertension was defined using thresholds of systolic blood pressure ≥160 mm Hg and diastolic blood pressure ≥100 mm Hg. In addition to blood pressure measurements, the investigators collected data on anthropometric indices and dietary practices, which indicated a mean BMI of 19.4 kg/m² and the notable absence of added salt to the diet, respectively. The findings revealed that only six men exhibited systolic blood pressure at or above 160 mm Hg and five had diastolic readings at or above 100 mm Hg. Moreover, unlike observations in American men, no significant age‐related increases in blood pressure were noted among the Maasai. These results suggest that hypertension was not a major cardiovascular risk factor in this population, likely due in part to their low body mass index, distinctive dietary practices, and high levels of physical activity [21,24].

Low serum cholesterol levels in the Maasai have also been reported. The study by Mann et al. (1964) assessed serum total cholesterol in participants by obtaining blood samples from each individual using evacuated tubes. The serum and plasma were frozen in dry ice and transported to a laboratory where serum total cholesterol was measured using an accurate and reproducible method detailed previously by the authors [32]. Among the 388 Maasai men examined, only eight were found to have serum cholesterol levels exceeding 200 mg/dL [24]. These observations were later confirmed by Biss et al. (1971), who reported a mean total cholesterol of 135 ± 34 mg/dL among 227 Maasai individuals over 15 years of age, with levels exceeding 200 mg/dL in only seven participants and without the significant age-related increases typical of populations in economically developed regions [26]. Although these findings indicate low cholesterol levels in the Maasai, it is noteworthy that potential confounders such as infections, which are discussed in detail in subsequent sections, may have influenced cholesterol levels.

Autopsy studies have also investigated the presentation of CVD among the Maasai. Biss et al. (1971) performed 10 consecutive autopsies on deceased Maasai, which indicated occasional fatty streaks in younger aortas and non-fatty intimal thickening or fibrous plaques in older aortas. Estimated ages and suggested causes of death were not described, but histochemical and chemical analyses demonstrated a marked paucity of atherosclerosis, and measurements of coronary arterial wall thickness demonstrated that the coronary arteries of the Maasai were significantly thinner than those of age- and sex-matched American men [26]. However, the small sample limits the generalizability of the results, underscoring the need for further research with larger cohorts to confirm these observations. A larger sample was used by Mann et al. (1972), who conducted autopsies on 50 Maasai men with estimated ages of death ranging from 10 to >60 years. The autopsies revealed extensive atherosclerosis with lipid infiltration and fibrous changes in the aorta. These findings were comparable to intimal thickening in the coronary arteries observed in older American men. Despite this evidence of atherosclerosis, the lesions rarely progressed to complications such as plaque rupture or thrombosis. The causes of death for the 50 men were combat (15), infection (13), cardiovascular or renal (8), accidents (6), malignancy (3), intestinal obstruction (2), suicide (2), and diabetic coma (1). The authors discussed potential reasons for the discrepant findings between the two autopsy studies and suggested that they may have been due to due to omissions of details in Biss et al. (1971) regarding subject selection, causes of death, evaluation methods, and age estimation [33].

Despite their preferable cardiovascular profile, early mortality relative to national averages has been reported in both historical and contemporary contexts for the Maasai [23]. High early-age mortality contributes significantly to this disparity but estimates also indicated that the life expectancy of a 20-year-old Maasai man was approximately 45 years [23]. This is significantly lower than the estimated life expectancy of 58 years for men residing in Kenya, based on the 1989 census [23]. Furthermore, a study of 436 Maasai men found that only three were over the age of 55 [24]. These factors complicate the interpretation of cardiovascular risk in the Maasai, as their reduced lifespan may prevent the manifestation of age-associated CVD typically seen in older populations. Unique biological adaptations and lifestyle factors appear to provide substantial protection against clinical CVD.

CVD-mitigating factors

Genetic Adaptations

The Maasai possess genetic adaptations that significantly influence their risk of diseases. These adaptations likely evolved under the combined pressures of the arid, parasite-rich environment and unique dietary patterns of the Maasai, which shaped their genetic makeup to optimize cholesterol metabolism and disease resistance [34]. Despite consuming diets rich in saturated fat and cholesterol, the Maasai have demonstrated low serum cholesterol levels, largely due to a highly efficient negative feedback mechanism that regulates endogenous cholesterol biosynthesis [26,35]. Metabolic studies have indicated that the Maasai absorb cholesterol at more than twice the rate of Caucasian Americans (650 vs. 300 mg/day), while simultaneously suppressing endogenous synthesis by 50%, compared to 25% in other populations [35]. Genetic analyses have identified several loci under selection in the Maasai, including those linked to cholesterol regulation, reflecting evolutionary responses to their high-cholesterol diet [16]. Collectively, these adaptations ensure that dietary cholesterol is efficiently managed, preventing hypercholesterolemia, and providing a physiological safeguard against the atherogenic effects of their dietary patterns [26]. Such genetic traits are rare and unique to populations like the Maasai, underscoring the importance of avoiding generalized dietary recommendations based on specific ethnic groups.

Calorie Restriction and Intermittent Fasting

The heightened food insecurity associated with the pastoral lifestyle of the Maasai has led to calorie restriction and intermittent fasting, each of which has implications for CVD risk. Food insecurity is reportedly high in northern Tanzania, but the Maasai have experienced more significant disadvantages than neighboring ethnic groups, with <5% of Maasai households reporting food security [36]. Energy intakes have been inadequate among the Maasai, ranging between 65 and 80% of recommended daily intakes (RDI) according to body weight, and 50-60% of the RDI according to age or physiological status [31,37]. Calorie restriction has been shown to improve cardiovascular risk factors [38]. For example, a meta-analysis including 32 randomized controlled trials investigating the effects of calorie restriction on cardiovascular risk found that one to four weeks of calorie restriction resulted in improvements in blood pressure comparable to those observed with commonly prescribed antihypertensive medications [39]. Furthermore, variable energy intakes and erratic meal patterns of gorging, followed by fasting, have characterized the Maasai diet [18]. Intermittent fasting, defined as alternating periods of eating and fasting, has been shown to improve an array of cardiovascular risk factors such as waist circumference, total cholesterol, LDL-cholesterol, triglycerides, and systolic blood pressure [40]. Together, these dietary practices may partially explain the low incidence of CVD among the Maasai.

Physical Activity and Energy Expenditure

The Maasai are highly physically active. A cross-sectional study including 130 Maasai men and women reported a mean energy expenditure of 2,565 kcal/day above basal requirements [21]. This approximates the energy expenditure of running a marathon [41]. High amounts of physical activity have been shown to mitigate the risk of developing or dying from heart disease [42], and it has been hypothesized that the high levels of physical activity in which the Maasai have engaged may help to compensate for dietary factors by promoting more capacious coronary arteries and cardiorespiratory fitness [21,33]. This level of physical activity is far from replicable in less physically active populations, where dietary patterns characterized by higher intakes of animal-based sources of fats and proteins are associated with an increased risk of CVD mortality [43-45]. Overlooking the role of exceptionally high levels of exercise in the Maasai lifestyle may therefore lead to inaccurate interpretations of their health outcomes, particularly when attempting to generalize their dietary patterns to less physically active populations.

Infections

The Maasai experience a high prevalence of infectious diseases and parasitic infections, which significantly influence their lipid profiles and CVD risk. Common diseases include malaria, tuberculosis, brucellosis, and gastrointestinal parasitic infections, which have been linked to the consumption of raw meat, milk, and blood, as well as poor sanitation and contaminated water [24,29,37,46,47]. These infections contribute to significant morbidity, with malaria affecting up to 35% of African pastoralists, and high reported prevalence of conditions including diarrhea and pneumonia among children [37,47]. Diarrhea and conjunctivitis were reported in approximately 20% and 40% of children, respectively [37]. Prevalent diseases in adults included treponemal infections and Brucellosis, which affected 25% and 50% of African pastoralists, respectively [37]. Infectious diseases have notable effects on lipid metabolism. For example, malaria and tuberculosis are associated with decreased levels of total cholesterol, HDL-cholesterol, and LDL-cholesterol compared to healthy controls, likely due to the metabolic demands of the diseases [48,49]. Mechanisms for altered blood lipids during infection include reduced synthesis of apolipoprotein A-I (apo A-I), increased production of serum amyloid A that binds to HDL and displaces apo A-I, and decreased lecithin cholesterol acyl transferase (LCAT) activity leading to impaired cholesterol esterification [50]. Treatment of tuberculosis restores lipid concentrations, highlighting the direct impact of infection on cholesterol levels [49]. Parasitic infections have been linked to increased HDL-cholesterol and reduced LDL-cholesterol levels, which may also influence CVD risk [51]. The significant burden of infections and their impact on lipid profiles may thereby complicate assessments of the relationships between diet, health, and cardiovascular risk in the Maasai population.

Altitude

The Maasai live at high elevations, such as the 2,000-meter altitude of the Maasai Mara [20]. Populations residing at high altitudes demonstrate improved lipid profiles, including higher levels of HDL-cholesterol, lower LDL-cholesterol, and reduced total cholesterol/HDL-cholesterol and LDL-cholesterol/HDL-cholesterol ratios [17,52]. These findings are attributed to hypoxia, which activates the hypoxia-inducible factor (HIF). The HIF upregulates pathways that promote HDL-cholesterol synthesis and enhance the clearance of triglyceride-rich lipoproteins by increasing lipoprotein lipase activity [53]. Concurrently, hypoxia inhibits sterol regulatory element-binding proteins, suppressing LDL production and hepatic cholesterol synthesis. These physiological adaptations contribute to reduced risk of metabolic syndrome and confer cardiovascular protection, as evidenced by lower coronary mortality in high-altitude populations [19,54]. Consequently, it has been recommended that elevation should be considered when assessing CVD risk across populations living at different altitudes [17].

Generalizability to meat-based diets

Health outcomes among the Maasai, often cited in support of meat-based diets, result from a unique combination of genetic, environmental, and lifestyle factors that are not replicable in other populations. Their traditional diet, which is rich in saturated fat and cholesterol, is paired with genetic adaptations that allow for efficient cholesterol metabolism, including a highly effective feedback mechanism that suppresses endogenous cholesterol synthesis [26,35]. This is not a trainable trait, in that individuals consuming diets high in fat and cholesterol in a modern setting would not be expected to adapt in a similar manner. Although these adaptations are well-documented among the Maasai, it remains uncertain whether similar genetic adaptations might be present in other populations in East Africa or elsewhere.

Furthermore, significant differences exist between the traditional Maasai diet and modern meat-based diets, with important implications for biomarkers of CVD risk. For example, although meat, animal fat, and dairy products are all included in the traditional Maasai diet, dairy products contribute 42% of total calories, whereas meat and animal fat intakes provide 9% of total calories in adult women [31]. The primary source of saturated fat in this diet is therefore dairy [28,31]. Modern carnivore diets also include meat and dairy, but meat is more central to this eating style while dairy intake is limited [55-57]. For example, red meat was reportedly consumed at each meal by 39% of carnivore diet adherents, whereas dairy milk was consumed with each meal by <1% of respondents [55]. This discrepancy has significant implications because dairy fat has been shown to increase LDL-cholesterol levels and CVD risk to a lesser extent than other sources of animal fat [58,59]. These differences are reflected in the serum cholesterol levels of these populations. Average serum total cholesterol levels in the Maasai have been reported to be 135 mg/dL [26]. In contrast, median serum total cholesterol levels in individuals following a modern carnivore diet were reported to be 256 mg/dL [55]. This discrepancy illustrates that the traditional diet and cardiovascular health profile of the Maasai are not generalizable to modern populations that follow meat-based diets. Notably, large-scale studies have demonstrated that, even after controlling confounding factors such as physical activity, smoking, and other lifestyle variables, increased consumption of red meat remains independently associated with higher risk of heart disease [60].

The health outcomes of populations are significantly influenced by their food environments, which differ substantially between undernourished pastoralist societies and industrialized nations. Environmental factors experienced by the Maasai such as periodic drought, marginalization, and land conflicts have led to high food insecurity and poor health outcomes [36]. This is reflected in a higher prevalence of underweight (BMI<18.5 kg/m^2^) among the Maasai (24%) compared to the Luo (14%) and Kamba (21%) ethnic groups in rural Kenya [61]. Similarly, children with height-for-age Z-scores (HAZ) <2 are classified by the World Health Organization as “stunted,” an indicator of long-term malnutrition, and significantly more Maasai children are stunted (57%) than children of the neighboring Meru (21%), Sukuma (32%), and Rangi groups (44%) [36]. A similar pattern is apparent with wasting, which has been recorded as significantly more prevalent in Maasai children (10%) compared to other neighboring ethnic groups (2-3%) [36]. The inclusion of both animal- and plant-sourced energy-dense foods has been shown to improve growth and health outcomes in such contexts by providing additional calories and micronutrients [62]. In contrast, industrialized nations are characterized by food environments that are abundant in energy-dense foods, including red and processed meats, which are associated with obesity and other biomarkers of chronic disease [63,64]. Conversely, greater intake of intakes of low-energy-dense plant-based foods, such as fruits and vegetables, is associated with lower risks of obesity and chronic diseases [65,66]. These contrasting observations highlight the contextual dependency of dietary patterns and emphasize the limitations of generalizing Maasai health outcomes, shaped largely by food scarcity, to populations in industrialized nations where caloric abundance prevails.

In addition to genetic and dietary factors, exceptionally high physical activity levels among the Maasai paired with intermittent fasting and caloric restriction, contribute to improved cardiovascular profiles [21,40]. High-altitude living further enhances lipid profiles through hypoxia-induced mechanisms, reducing CVD risk [19,53]. These protective factors differ significantly from the sedentary lifestyles and caloric excess prevalent in industrialized societies. Moreover, low rates of clinical CVD among the Maasai are influenced by high rates of infectious diseases, which modify lipid profiles, and their reduced life expectancy, which prevents many from reaching the age at which CVD typically manifests [24,49].

## Conclusions

The health outcomes of the Maasai are often used to promote meat-based diets but cannot be understood solely through the lens of their traditional dietary practices. Their low rates of CVD events are shaped by a complex combination of factors, including unique genetic traits for cholesterol metabolism, high levels of physical activity, calorie restriction, intermittent fasting, high-altitude living, and a significant burden of infectious diseases. These elements, combined with relatively short life expectancy among the Maasai, underscore the limitations of generalizing their health outcomes to modern, less physically active populations consuming meat-rich diets that differ vastly in composition and context. By oversimplifying the Maasai health profile, current narratives risk perpetuating misconceptions about the benefits of high-meat diets for broader populations. This highlights the need for dietary guidelines that consider the broader socio-ecological context, as well as the genetic, environmental, and lifestyle factors that are unique to the target population. Such an approach ensures not only scientific rigor but also practical relevance and efficacy in promoting public health across different populations.
